# Functional Conservation of Divergent p63-Bound *cis*-Regulatory Elements

**DOI:** 10.3389/fgene.2020.00339

**Published:** 2020-04-29

**Authors:** Lourdes Gallardo-Fuentes, José M. Santos-Pereira, Juan J. Tena

**Affiliations:** Centro Andaluz de Biología del Desarrollo (CABD), Consejo Superior de Investigaciones Científicas/Universidad Pablo de Olavide, Seville, Spain

**Keywords:** p63, transcription factor binding sites, functional conservation, sequence divergence, human, zebrafish

## Abstract

The transcription factor p63 is an essential regulator of vertebrate ectoderm development, including epidermis, limbs, and craniofacial tissues. Here, we have investigated the evolutionary conservation of p63 binding sites (BSs) between zebrafish and human. First, we have analyzed sequence conservation of p63 BSs by comparing ChIP-seq data from human keratinocytes and zebrafish embryos, observing a very poor conservation. Next, we compared the gene regulatory network orchestrated by p63 in both species and found a high overlap between them, suggesting a high degree of functional conservation during evolution despite sequence divergence and the large evolutionary distance. Finally, we used transgenic reporter assays in zebrafish embryos to functionally validate a set of equivalent p63 BSs from zebrafish and human located close to genes involved in epidermal development. Reporter expression was driven by human and zebrafish BSs to many common tissues related to p63 expression domains. Therefore, we conclude that the gene regulatory network controlled by p63 is highly conserved across vertebrates despite the fact that p63-bound regulatory elements show high divergence.

## Introduction

Mutations affecting the activity of *cis*-regulatory sequences are thought to be the most prevalent cause of phenotypic divergence in animal evolution (Carroll, 2008; Stern and Orgogozo, 2008). Given that promoter sequences are bound by a collection of highly conserved and widely used transcriptional regulators, they do not seem to be the main driver of *cis*-regulatory divergence (Brown and Feder, 2005). Indeed, enhancers are usually more variable between species, and they are the type of *cis*-regulatory element (CRE) thought to be accountable for *cis*-regulatory divergence (Wray, 2007). Thus, enhancers are potential targets for evolutionary change since they modulate target gene expression in specific tissue contexts and ordinarily exist in groups of redundant elements, making easy the accumulation of genetic modifications by dampening the risk of lethality (Levine, 2010; Wittkopp and Kalay, 2011).

Alterations in CRE sequences will affect *cis*-regulatory activity through the modification of transcription factor (TF) binding sites (BSs) (Wilson et al., 2008; Zheng et al., 2010) since they are the functional units of *cis*-regulatory sequences. Possible changes in TF binding are gains or losses of BSs or changes in the affinity of the site (Borneman et al., 2007; Odom et al., 2007; Tuch et al., 2008; Schmidt et al., 2010), although the latter possibility could have a minor contribution since TF binding specificities are highly conserved (Nitta et al., 2015).

p63 is a TF of the p53 family and an essential regulator of ectoderm development, including epidermis, limbs, and craniofacial tissues (Soares and Zhou, 2018). Heterozygous mutations in the *TP63* human gene are associated with several hereditary malformations showing ectrodactyly (split hand or foot malformations), ectodermal dysplasia, and orofacial cleft as their principal phenotypes (Celli et al., 1999; Rinne et al., 2006). Null or knockdown animal models exhibit severe limb truncations, as well as absence of skin and derived tissues (Yang et al., 1998; Mills et al., 1999; Bakkers et al., 2002; Lee and Kimelman, 2002; Santos-Pereira et al., 2019).

In this work, we have explored the evolutionary conservation of p63 BSs between zebrafish and human. For this, we have first analyzed the sequence conservation of p63 BSs of human keratinocytes and zebrafish embryos, showing a very poor conservation; however, about one third of the BS-associated genes are orthologous between these two species, suggesting that the p63 gene regulatory network is conserved. Indeed, we validated this functional conservation using transgenic reporter assays in zebrafish and found that diverged enhancers with species-specific p63 binding from both species drove similar expression patterns. Altogether, our data suggest that the gene regulatory network regulated by p63 is conserved across vertebrates and despite the fact that CRE sequences diverged along species evolution.

## Materials and Methods

### Animal Experimentation

All experiments involving animals conform to the national and European Community standards for the use of animals in experimentation and were approved by the Ethical Committees from the University Pablo de Olavide, Consejo Superior de Investigaciones Científicas (CSIC), and the Andalusian government.

### Zebrafish Husbandry

Wild-type fishes were crossed for 10 min roughly since first eggs laid to obtain synchronous embryos. The eggs were collected in petri dishes with E3 medium (5 mM NaCl, 0.17 mM KCl, 0.33 mM CaCl_2_, 0.33 mM MgSO_4_, and 0.1% methylene blue). Wild-type strains for zebrafish were AB/Tübingen (AB/Tu) and were maintained and bred under standard conditions (Westerfield, 2007). Embryo stages were expressed in hours post-fertilization (hpf) as described (Kimmel et al., 1995).

### Transgenic Reporter Assays

For enhancer cloning, zebrafish and human genomic fragments containing the studied CREs were amplified with iMAX-II DNA Polymerase (Intron Biotechnology) using primers from Supplementary Table S1. The PCR fragments were purified using Isolate II PCR and Gel Kit (BIOLINE), sub-cloned in the pCR8/GW/TOPO vector (Invitrogen) and then transferred, through recombination using Gateway LR technology (Invitrogen), to the enhancer detection vector for zebrafish transgenesis, containing the strong midbrain enhancer z48 and the green fluorescent protein (*GFP*) reporter gene under the control of the *gata2* minimal promoter (Gehrke et al., 2015).

Zebrafish transgenic embryos were generated using the Tol2 method (Kawakami et al., 2004). One-cell-stage embryos were injected with 3–5 nl of a solution containing 30 ng/μl of Tol2 mRNA, 20 ng/μl of phenol:chloroform-purified enhancer detection vector, and 0.05% of phenol red solution. Injected embryos (F0) were selected for GFP expression at 48 hpf, raised to sexual maturity and screened for germline transmission. GFP-expressing F1 embryos were photographed at 24 and 48 hpf stages with a digital CCD camera (MagnaWre, Optronix) mounted on an MZ-12 dissecting scope (Leica). Three independent stable transgenic lines showing similar GFP expression patterns were generated.

### ChIP-Seq Data Analyses

ChIP-seq data of human keratinocytes (Kouwenhoven et al., 2010) and ChIPmentation data of 24-hpf zebrafish embryos (Santos-Pereira et al., 2019) were obtained from datasets GSE17611 and GSE123057, respectively. Reads were aligned to the GRCh37/hg19 human genome assembly or the GRCz10/danRer10 zebrafish genome assembly using Bowtie (Langmead et al., 2009). Peaks were called using MACS2 algorithm (Zhang et al., 2008) with a false discovery rate (FDR) < 0.001, and common peaks to all biological replicates, calculated with the Intersect tool from the Bedtools package (Quinlan and Hall, 2010), were considered as high-confidence peaks for each species. The University of California–Sta. Cruz (UCSC) Genome Browser was used to visualize ChIP-seq data (Casper et al., 2018). TF motif enrichment was calculated using the script FindMotifsGenome. pl from Homer software (Heinz et al., 2010) with standard parameters.

For gene assignment to ChIP-seq peaks, zebrafish coordinates were converted to Zv9/danRer7 genome using the Liftover tool of the UCSC Genome Browser (Casper et al., 2018). Both zebrafish and human peaks were assigned to genes using the GREAT tool (Hiller et al., 2013), with the basal plus extension association rule with standard parameters (5 Kb upstream, 1 Kb downstream, 1 Mb maximum extension). Ensembl BioMart tool (Zerbino et al., 2018), release 75 for GRCh37/hg19 and release 91 for GRCz10/danRer10, was used to obtain the orthologous genes in human and zebrafish. The significance of the overlaps between lists of orthologous genes was assessed using the hypergeometric test and the number of total orthologous genes for each species as reference. PANTHER (Mi et al., 2017) was used to calculate Gene Ontology (GO) terms overrepresented. For sequence conservation analyses, genomic coordinates of the p63 BSs in human were converted to zebrafish coordinates, and vice versa, using the Liftover tool from the UCSC Genome Browser (Casper et al., 2018).

## Results

### Poor Sequence Conservation of p63 Binding Sites Between Zebrafish and Human

**FIGURE 1:**
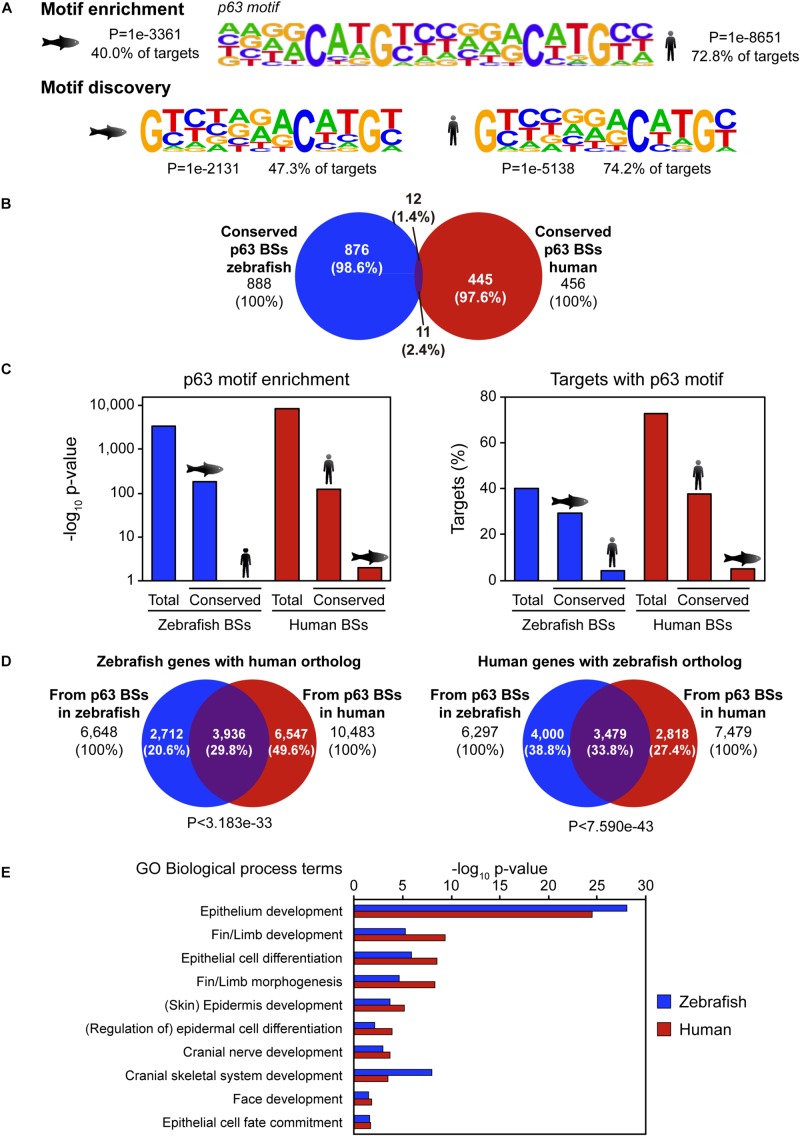
Sequence divergence but functional conservation of p63 binding between zebrafish and human. **(A)** p63 motif logo found by either motif enrichment (top) or *de novo* motif discovery (bottom) in p63 ChIP-seq from zebrafish embryos (left) and human keratinocytes (right). Enrichment *p*-value and percentage of targets with the motif are represented. **(B)** Venn diagram showing the overlap between p63 binding sites (BSs) in zebrafish and human conserved at the sequence level. **(C)** p63 motif enrichment (left) and percentage of targets with p63 motif (right) for total and conserved p63 BSs in zebrafish and human. **(D)** Venn diagrams showing the overlap between zebrafish p63-associated genes with human ortholog (left) or human p63-associated genes with zebrafish ortholog (right) from BSs in both species. *P*-values for overlap significance using the Hypergeometric test are shown. **(E)** Gene Ontology (GO) biological process term enrichment for zebrafish and human p63 BSs. Terms related to p63 known functions were selected. Total GO terms are shown in Supplementary Dataset.

p63 is a conserved master regulator of vertebrate ectoderm development. Thus, we wondered whether its BSs were also conserved. To address this question, we took advantage of published ChIP-seq data of p63 in human keratinocytes (Kouwenhoven et al., 2010) and compared them with our ChIP-seq data in zebrafish 24-hpf embryos (Santos-Pereira et al., 2019). Peak calling identified 11,466 high confidence p63 BSs in human keratinocytes and 10,520 in zebrafish embryos. *De novo* motif discovery analysis identified a p53-like binding sequence that was virtually the same in both species despite their high evolutionary divergence (Figure 1A). These motifs represent indeed incomplete versions of the p63 motif described for human keratinocytes (Kouwenhoven et al., 2010), which is also enriched in the p63 peaks called in both species (Figure 1A).

We hypothesized that the main CREs involved in p63 regulatory network may be highly conserved at the sequence level. In order to check this possibility, we analyzed sequence conservation between p63 BSs in zebrafish and human and found 888 BSs in zebrafish and 456 BSs in human keratinocytes falling into conserved regions (8.44 and 3.98% of total p63 BSs, respectively; Figure 1B). Next, we analyzed which of those conserved BSs corresponded to p63 BSs in the other species. For this, we intersected the 456 conserved human BSs with the 888 conserved zebrafish p63 BSs, getting as few as 11 human and 12 zebrafish BSs (2.4 and 1.4% of total, respectively; Figure 1B). Although human data comes from cultured keratinocytes and zebrafish samples come from whole embryos, the embryonic epidermis represents a large proportion of the whole embryo, and therefore the results obtained in both systems should be, at some extent, comparable. Taking this into account, these results indicate that most p63 BSs do not have sequence conservation between zebrafish and human.

In order to assess whether the absence of protein binding to conserved BSs in both species was due to a loss of affinity of p63 for these BSs, we analyzed the enrichment of the human and zebrafish p63 motifs (Figure 1A) in these sites. We observed that the p63 motif was highly enriched in the zebrafish conserved BSs, but not in their human orthologous regions. Conversely, the p63 motif was highly enriched in the human conserved BSs, but not in their zebrafish counterparts (Figure 1C). This result indicates that, despite the high degree of conservation of this subset of p63 BSs between the two species, these sequences have diverged enough to lose the p63 binding motif.

### The p63 Gene Regulatory Network Is Highly Conserved Between Zebrafish and Human

Given the absence of sequence conservation of p63 BSs, we wondered whether the gene regulatory network controlled by p63 was conserved even though the CREs’ sequence have diverged between species. For this, we used the GREAT tool to assign putatively regulated genes to the p63 BSs of each species. Thus, the 10,520 p63 BSs in zebrafish were associated with 7,547 genes, while the 11,466 p63 BSs in human were associated with 8,738 genes. In order to compare genes in both species, we selected the BS-associated genes showing at least one ortholog in the other species. This analysis was performed in both directions, i.e., starting either from zebrafish or human BS-associated genes. We found 6,648 zebrafish genes associated with zebrafish p63 BSs that corresponded to 6,297 human orthologs. On the other hand, we found 7,479 human genes associated with keratinocyte p63 BSs that corresponded to 10,483 zebrafish genes (note that the zebrafish genome shows more than one ortholog to many human genes due to the additional whole genome duplication of teleost fishes).

Then, we analyzed the overlap between orthologous genes associated with p63 ChIP-seq data in both species and found that 29.8% of zebrafish genes with human orthologs and 33.8% of human genes with zebrafish orthologs overlapped (*P* < 3.183e-33 and *P* < 7.590e-43, respectively) (Figure 1D). In other words, one third of the p63 target genes were conserved between both species. Next, by means of GO term enrichment analyses, we checked whether the biological functions of these conserved genes were the same in both species. These GO analyses showed many biological processes related to p63 functions, among other developmental functions, associated with the conserved genes. These included “skin/epidermis development,” “fin/limb development,” or “face development” (Figure 1E and Supplementary Dataset). However, these p63-related GO terms were absent for the species-specific genes, with the exception of “skin development” for the human orthologs of zebrafish-specific genes (FDR < 1.13e-3). In these species-specific genes, GO terms related to metabolic functions and olfactory perception were among the most enriched ones (Supplementary Dataset). Altogether, our data show that the p63 gene regulatory network is conserved across vertebrates despite the sequence divergence observed at the CRE level.

### Functional Conservation of Vertebrate p63 Binding Sites

The observation that p63 BSs have diverged between zebrafish and human at the sequence level, but their associated genetic network is highly conserved, prompted us to seek for non-conserved p63 BSs driving similar expression patterns of orthologous genes in zebrafish and human. For this, we selected a set of six pairs of p63 BSs from both species that were putatively targeting the same epidermal genes and located in equivalent positions in both genomes: upstream of the *dlx3b/DLX3, grhl1/GRHL1*, and *myh9a/MYH9* zebrafish/human genes, and in the introns of *st14a/ST14, lama5/LAMA5*, and *map2k1/MAP2K1*. Then, these zebrafish and human sequences were cloned into an enhancer detection vector composed of a *gata2* minimal promoter, an enhanced *GFP* reporter gene, and the strong midbrain enhancer z48 (Gehrke et al., 2015). Thus, after screening for F0 founder individuals, the expression patterns driven by these CREs could be followed by monitoring GFP expression in F1 embryos.

**FIGURE 2:**
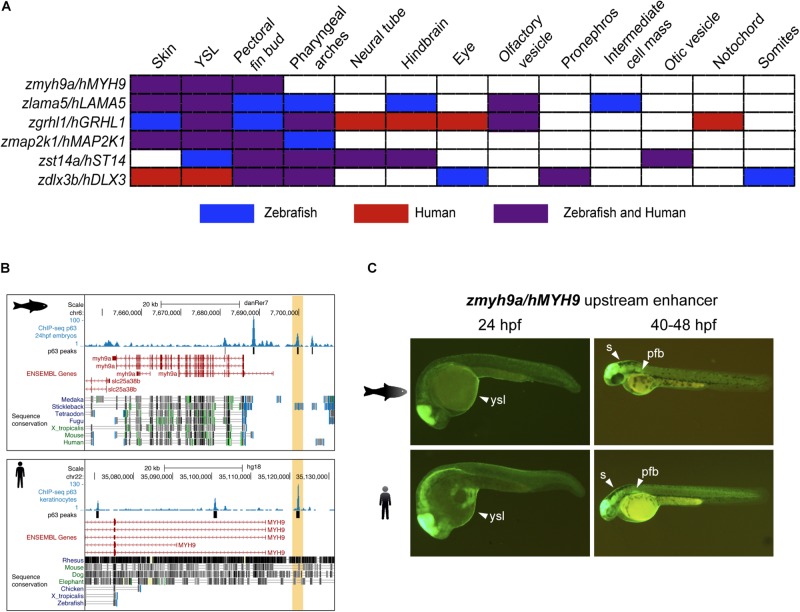
Zebrafish and human p63 binding sites (BSs) drive similar expression patterns. **(A)** Table showing the overlap in expression patterns driven by *cis*-regulatory element (CRE) pairs from zebrafish and human, as detected in zebrafish transgenic reporter assays. Note that all BS pairs share at least three expression domains in both species. **(B)** Genome tracks from zebrafish (top) and human (bottom) showing p63 ChIP-seq signal, p63 peaks, ENSEMBL genes, and vertebrate sequence conservation. The selected p63 BSs for transgenic reporter assays, which are located upstream to the *myh9a/MYH9* genes, are highlighted in light orange. **(C)** Transgenic zebrafish embryos at different stages showing the green fluorescent protein (*GFP*) expression pattern driven by the enhancers highlighted in **(B)**. Tissues expressing GFP are pointed using white arrowheads. *s*, skin; *ysl*, yolk syncytial layer; *pfb*, pectoral fin bud.

We analyzed the expression driven by these CREs in 24 and 48 hpf zebrafish F1 embryos and found partial to total overlapping tissues for all the CRE couples (Figure 2A). Interestingly, we found that the p63 BSs located upstream to the *myh9a/MYH9* genes drove GFP expression to typical p63-expressing tissues in both species, including the skin, the yolk syncytial layer (YSL), and the pectoral fin bud (Figures 2B,C). This pattern is highly consistent with the expression of the zebrafish *myh9a* gene in epidermis, periderm, and pectoral fin bud (Huang et al., 2013). For other CREs, we found overlapping GFP expression in additional p63-expressing tissues, such as the pharyngeal arches, but also in neural tissue, such as hindbrain and neural tube, sensory organs, such as the eyes and olfactory and otic vesicles, and even in mesodermal lineages, such as the inner cellular mass, pronephros, somites, and notochord for specific cases (Supplementary Figure S1).

The expression patterns driven by these other p63 BSs were also totally or partially consistent with the expression of the zebrafish genes that are putatively controlled by them. For instance, the *lama5* gene has been described to be expressed in the epidermis, apical ectodermal ridges at the pectoral fin buds, lens, midbrain–hindbrain boundary, and pronephros^1^, consistent with expression driven by the analyzed p63 BSs in skin, YSL, pectoral fin buds, and hindbrain. Also, the gene *grhl1* is expressed in the epidermis, periderm, enveloping layer (EVL), olfactory placodes, otic placodes and vesicle, pharyngeal arches, and pronephros (Jänicke et al., 2010), while the analyzed p63 BSs drove reporter expression to the skin, YSL, pectoral fin buds, pharyngeal arches, and olfactory vesicle, as well as the hindbrain, neural tube, eyes, and notochord specifically for the human BS. The gene *dlx3b* is expressed in the pectoral fin bud, median fin fold, otic vesicle, and pharyngeal arches^1^, while p63 BSs from both species lead to consistent expression in the pectoral fin buds, pharyngeal arches, and pronephros. For the gene *st14a*, little overlap is found between the gene expression patterns (Carney et al., 2007) and those driven by the analyzed p63 BSs, although the overlap between BSs from both species is consistent. Finally, no previous information of expression patterns is available for the *map2k1* gene.

Altogether, these data suggest that the expression of p63 target genes has been conserved in vertebrates through a rewiring of the enhancers containing p63 BSs.

## Discussion

In this work, we have studied the sequence and functional conservation of genomic BSs of the epidermal TF p63 between human and zebrafish. We observe that most p63 BSs in one species are not conserved at the sequence level in the other species, and that the conserved BSs do not reproduce p63 binding nor p63 motif enrichment in the other species (Figure 1). However, there is a high degree of overlap of the ortholog genes associated with p63 BSs (Figure 1), suggesting conservation of the gene regulatory network across vertebrates despite the large evolutionary distance and the observed sequence divergence. Finally, we have functionally validated this conservation by transgenic reporter assays in zebrafish embryos showing overlapping expression patterns driven by selected BSs from both species associated with common epidermal genes (Figure 2 and Supplementary Figure S1).

Previous studies using transgenesis assays with human and zebrafish developmental enhancers (Fisher et al., 2006; Rada-Iglesias et al., 2011; Taher et al., 2011) or with human and mouse heart enhancers (Blow et al., 2010; May et al., 2012) showed a high degree of functional conservation despite sequence divergence, which is in agreement with our findings. In addition, a computational comparative analysis of p63 BSs in human and mouse keratinocytes found a higher sequence conservation between both species since they diverged more recently, although many of the conserved BSs lost p63 binding and p63 motif enrichment in the other species (Sethi et al., 2017), suggesting an evolutionary rewiring of the p63-associated CREs that is also consistent with our results. In this study, they also found that common p63 BSs putatively control a core network of epithelial genes, while species-specific genes are enriched for metabolic functions (Sethi et al., 2017). Some authors suggest that TF BS turnover is a mechanism that could explain this phenomenon, i.e., the loss of a TF BS can be compensated by the gain of a new BS for the same TF. This can occur directly by changes in the genome sequence involving the lost or gained BSs (Schmidt et al., 2010; Xie et al., 2010) or by insertions of complete *cis*-regulatory modules by transposable elements (Kunarso et al., 2010; Xie et al., 2010). In any case, we propose that this could be the case of p63 BSs, for which BS losses may have been compensated by BS gains close to the same target genes, leading to divergence of the original sequence due to the absence of selective pressure, but maintaining the control over a common subset of developmental genes.

## Data Availability Statement

Publicly available datasets were analyzed in this study. This data can be found here: GSE17611 and GSE123057.

## Ethics Statement

The animal study was reviewed and approved by Comité de Ética de la Universidad Pablo de Olavide (Seville).

## Author Contributions

JT and JS-P conceived and designed the project. LG-F performed the experiments. LG-F, JS-P, and JT analyzed the data and wrote the manuscript.

## Conflict of Interest

The authors declare that the research was conducted in the absence of any commercial or financial relationships that could be construed as a potential conflict of interest.

## Footnotes

1 http://zfin.org
