# Supplementary material for: Functional Conservation of Divergent p63-Bound cis-Regulatory Elements
Source: Front Genet. 2020 Apr 29;11:339. doi: 10.3389/fgene.2020.00339 (PMC7200997; doi:10.3389/fgene.2020.00339)
Supplement: Supplementary file 2 [file Data_Sheet_2.PDF]

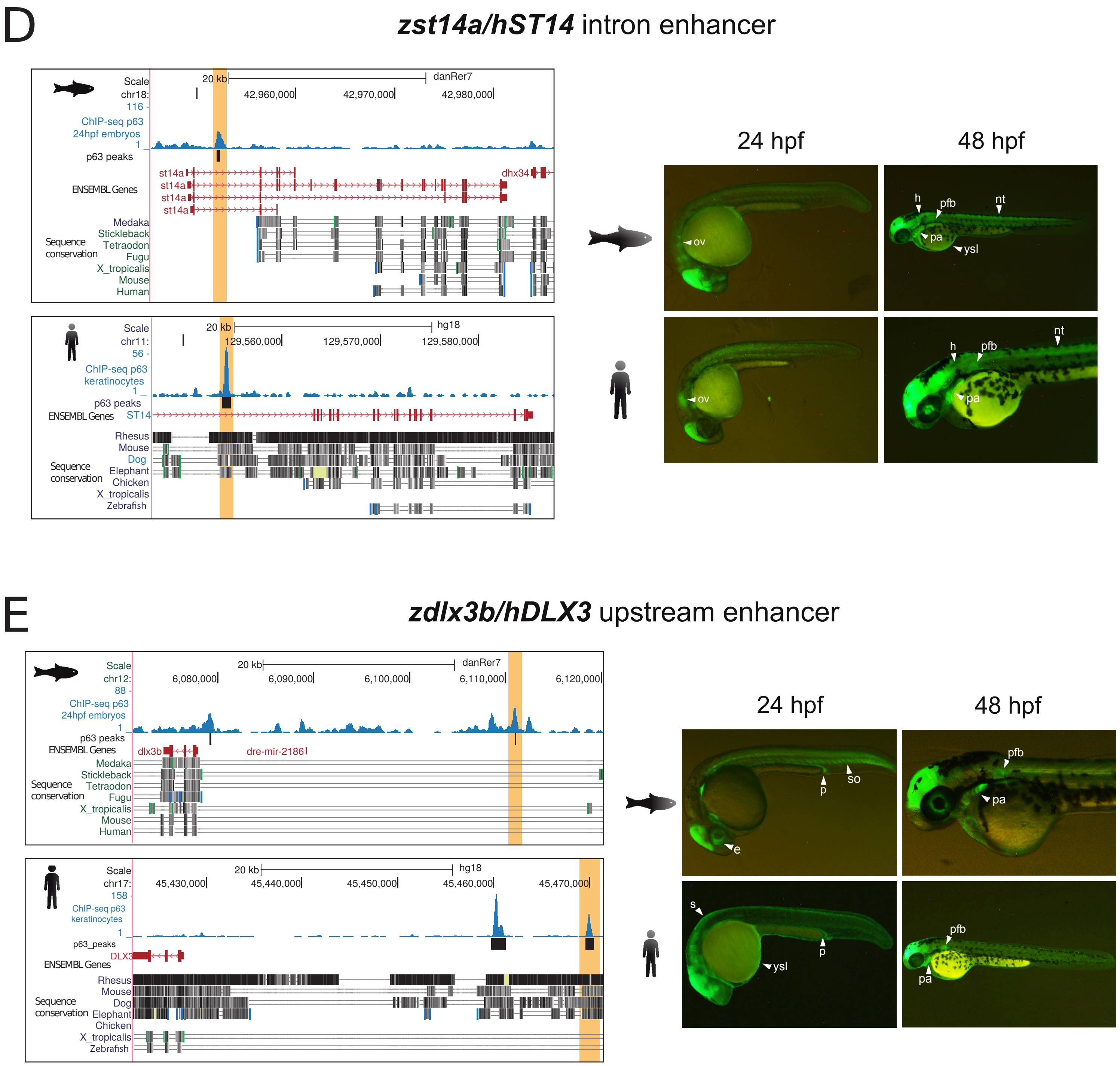

Supplementary Figure S1 – Reporter assays in zebrafish embryos with equivalent zebrafish and human p63 binding sites. Several couples of equivalent p63 binding sites from zebrafish and human were assayed: (**A**) *zlama5/hLAMA5* intron enhancer, (**B**) *zgrhl1/hGRHL1* upstream enhancer, (**C**) *zmap2k1/hMAP2K1* intron enhancer, (**D**) *zst14a/hST14* intron enhancer and (**E**) *zdlx3b/hDLX3* upstream enhancer. *Left*, genome tracks from zebrafish (top) and human (bottom) showing p63 ChIP-seq signal, p63 peaks, ENSEMBL genes and vertebrate sequence conservation. *Right*, transgenic zebrafish embryos at 24 and 48 hpf showing the *GFP* expression pattern driven by the analyzed enhancers. s, skin; yls, yolk syncytial layer; pfb, pectoral fin bud; pa, pharyngeal arches; nt, neural tube; h, hindbrain; e, eye; olv, olfactory vesicle; p, pronephros; icm, intermediate cell mass; ov, otic vesicle; n, notochord; so, somites.
