# Supplementary material for: Functional Conservation of Divergent p63-Bound cis-Regulatory Elements
Source: Front Genet. 2020 Apr 29;11:339. doi: 10.3389/fgene.2020.00339 (PMC7200997; doi:10.3389/fgene.2020.00339)
Supplement: Supplementary file 3 [file Table_1.DOCX]

**Supplementary Table S1 -** Sequences of primer oligonucleotides used to amplify the p63 binding sites (BS). Those that start with “z” correspond to the zebrafish BS, and those that start with “h” correspond to the human BS. “up” means upstream regarding the gene orientation and “i” means intron.

| **Enhancer** | **Forward** | **Reverse** | **Size (bp)** |
| --- | --- | --- | --- |
| zdlx3b-up | TTCAATGCAAATGTGTCTCC | TTTGGAGTGTGTTGTAGCC | 653 |
| zgrhl1-up | TACACTCACTCTCTCTCTCG | ACAGCCTGATAACATCTTCC | 717 |
| zmyh9a-up | TGCAGCTAAATATCAACTTCG | AATCAGTGCTCATATCTGGC | 537 |
| zst14a-i | GAAACAGAAACGGTTGGC | ACATGACATCCTTCTTGACC | 533 |
| zlama5-i | TTCATAGTACAAGGGTGAGG | CTAGGAAACCATAGCAGACG | 994 |
| zmap2k1-i | CTACCTTTATTTGGCAAGCC | ACAAATGGAAACTTGCTTCG | 633 |
| hdlx3-up | GTCATGGAGGCAGAGTCG | TTACTCATGTCTCACTCAGG | 639 |
| hgrhl1-up | CAGTTTGGTTTAACAGGTGC | ATATCTTCATCGCCATCTCC | 800 |
| hmyh9-up | AAACTGGGTTGTTGTGGG | CTGAGACTGCATTTCTAAAGG | 559 |
| hst14-i | CTGGAGTGTGTATTTCTGGG | CTGAGAATGATACCAGAGGC | 835 |
| hlama5-i | ATTGCTGGCTGATGACATGG | GCTCAGACAGGACAAAGG | 591 |
| hmap2k1-i | GAGTGGCTCATTAGTAGTGG | TTTGTGATCTGAGGAGGAGG | 817 |
