# Supplementary material for: Functional Conservation of Divergent p63-Bound cis-Regulatory Elements
Source: Front Genet. 2020 Apr 29;11:339. doi: 10.3389/fgene.2020.00339 (PMC7200997; doi:10.3389/fgene.2020.00339)
Supplement: Supplementary file 1 [file Data_Sheet_1.PDF]

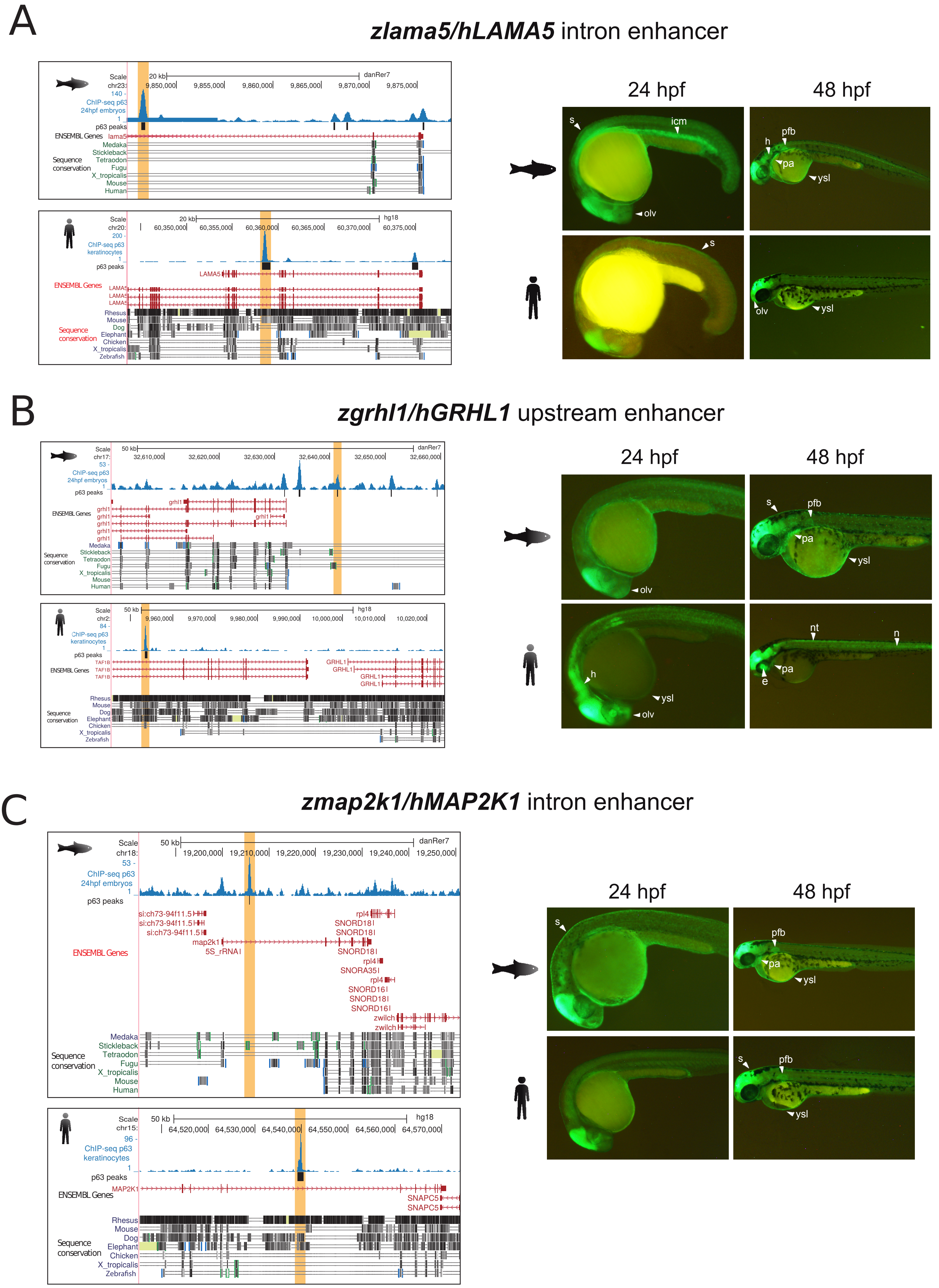
A
zlama5/hLAMA5 intron enhancer
24 hpf
48 hpf
Scale
chr23:
danRer7
ChIP-seq p63
24hpf embryos
p63 peaks
ENSEMBL Genes
lama5
Sequence conservation
Medaka
Stickleback
Tetraodon
Fugu
X_tropicalis
Mouse
Human
chr20:
hg18
ChIP-seq p63
keratinocytes
LAMA5
Rhesus
Dog
Elephant
Chicken
Zebrafish
s
icm
olv
h
pfb
pa
ysl
B
zgrhl1/hGRHL1 upstream enhancer
24 hpf
48 hpf
chr17:
grhl1
chr2:
TAF1B
GRHL1
nt
n
e
C
zmap2k1/hMAP2K1 intron enhancer
24 hpf
48 hpf
chr18:
map2k1
5S_rRNA
rpl4
SNORD18
SNORA35
SNORD16
zwilch
chr15:
MAP2K1
SNAPC5
